# Protocol for fabricating elastomeric stencils for patterned stem cell differentiation

**DOI:** 10.1016/j.xpro.2024.103187

**Published:** 2024-11-26

**Authors:** Stefanie Lehr, Jack Merrin, Monika Kulig, Thomas George Minchington, Anna Kicheva

**Affiliations:** 1 Institute of Science and Technology Austria, Am Campus 1, 3400 Klosterneuburg, Austria

**Keywords:** Developmental biology, Cell Differentiation, Organoids, Biotechnology and bioengineering

## Abstract

Geometrically controlled stem cell differentiation promotes reproducible pattern formation. Here, we present a protocol to fabricate elastomeric stencils for patterned stem cell differentiation. We describe procedures for using photolithography to produce molds, followed by molding polydimethylsiloxane (PDMS) to obtain stencils with through holes. We then provide instructions for culturing cells on stencils and, finally, removing stencils to allow colony growth and cell migration. This approach yields reproducible two-dimensional organoids tailored for quantitative studies of growth and pattern formation.

For complete details on the use and execution of this protocol, please refer to Lehr et al.^1^

## Highlights

• Production of PDMS stencils with predefined patterns
• Using stencils to initialize stem cell colony formation with controlled geometry
• Subsequent stencil removal allows unconstrained colony growth and cell migration
• Suitable for quantitative analysis of self-organized patterns and growth

## Abstract (editor highlights)

Publisher’s note: Undertaking any experimental protocol requires adherence to local institutional guidelines for laboratory safety and ethics.

## Graphical abstract

**Figure:**
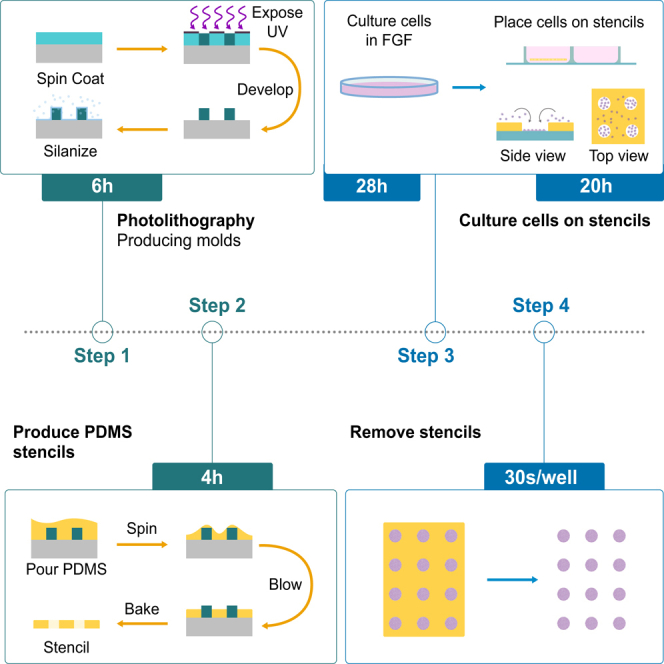

## Before you begin

### Background and motivation

The directed differentiation of embryonic stem cells (ESC) into defined cell types has had a marked impact on tissue engineering and on studies of embryonic development and disease. In recent years, it has become increasingly clear that geometric constraints are essential to overcome the intrinsic heterogeneity of *in vitro* differentiation systems.^2^^,^^3^^,^^4^ This principle has been applied to two-dimensional ESC differentiation systems on restricted micropatterns to yield quantitative data on pattern formation and signaling, for instance during mouse and human gastrulation.^5^^,^^6^^,^^7^ Micropatterned cell culture surfaces are created by defining areas that promote cell attachment, passivated areas to prevent cell attachment, or some combination of the two.^8^^,^^9^^,^^10^ While these systems provide a convenient quantitative readout of pattern formation, the confinement of cells within a restricted surface limits the applicability of this approach to investigating growing tissues and migratory cell populations.

We recently developed a method for directed differentiation of mouse ESCs into cell types of the developing dorsal spinal cord.^1^ In contrast to other protocols that derive dorsal neural progenitors from embryoid bodies,^11^^,^^12^ this method is based on a monolayer differentiation of neuromesodermal progenitor cells^13^ that are subsequently directed towards posterior dorsal neural tube fates. With this approach, cell types of the dorsal spinal cord form remarkable self-organized patterns upon exposure to BMP4. Trunk neural crest, a highly migratory cell type, forms at the periphery, followed by roof plate and dorsal neural progenitor subtypes dp1-6 towards the center of colonies in their correct spatial order.^1^ To allow for neural crest migration and reproducible two-dimensional patterning of the colonies, we use an approach that relies on removable PDMS microwell stencils.^14^^,^^15^ This initializes colony formation on a defined area and subsequently allows colony growth and migration. Our approach contrasts with previous methods that use microcontact printing or photo-patterning to passivate surface areas in a manner that cannot be easily reversed.^10^ By providing a removable barrier to colony growth, rather than modifying the substrate, the stencil method offers the flexibility to use a geometric constraint only transiently during the experiment.

Here, we provide an extended step-by-step protocol for stencil microfabrication adapted for the directed differentiation of mouse embryonic stem cells into dorsal spinal neural tube progenitors.

***Note:*** Stencil fabrication using this protocol will require expertise in photolithography and standard approaches for working with PDMS for soft lithography. Typically, this involves the use of a cleanroom. ESC differentiation requires cell culture experience and a dedicated cell culture room.

### Design and ordering of the photomask

**Timing:** 1**–4 weeks**

**Figure 1:**
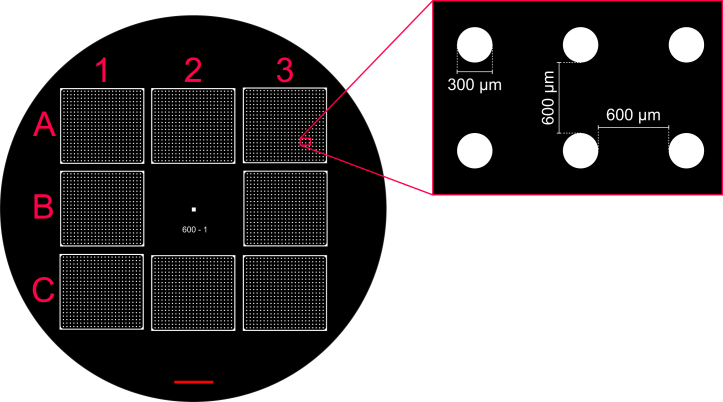
Photomask design Diagram of the design of a photomask suitable for the round geometry of the silicon wafers (Siegert wafer) for 8 stencils with dimensions 21.3 × 19.1 mm^2^ each. The zoomed window shows the spacing of the micropattern with 300 μm holes separated by 600 μm spaces. Black areas represent opaque areas of the mask which match the shape of the wafer, while white areas allow UV exposure of the photoresist and will form the pegs of the mold. Red letters and numbers are used to indicate the positions of the individual stencils in the grid. The photomask shown here is an example; the design should be tailored to the specific experiment. Scale bar, 10 mm.

***Note:*** Photomasks are ordered from an external company; therefore, this step has to be completed in advance. We ordered photomasks from JD Photo Data (UK) as a 9″ × 12″ film photomask at the highest available resolution of approximately 10 μm with positive polarity (i.e., transparent areas define the pattern; see Figure 1).

The photomask with the desired pattern can be designed using CAD software and ordered from a specialist supplier, and is used to fabricate a master silicon wafer mold for the stencils using photolithography. The transparent areas on the mask will become posts in the mold and, therefore, define the holes in the final stencils (Figure 1).

1. Check with your photomask manufacturer to ensure file type compatibility. We use LinkCad to convert files from dxf to Gerber format.
2. The photomask design comprises a 3 × 3 array of rectangular stencils, each 21.3 × 19.1 mm^2^ (to fit the dimensions of 2-well ibidi slides) with a space in the center (Figure 1). This design can produce eight stencils. Up to 4 of these designs (4″ × 4″) can be ordered on a single 9″ × 12″ transparency.
3. The photomask pattern for each stencil was designed such that there are 483 holes with a diameter of 300 μm. The holes are separated by a 600 μm spacing and arranged in a rectangular lattice (Figure 1).
4. The center space was used for labeling but also contains a square of 1 × 1 mm^2^ in the center, which will become a post for checking the height precision of the mold after manufacture.

### Preparation of mouse embryonic stem cells

**Timing: ∼1 week**

This step describes the preparation of mouse ESCs to use for differentiation. Cells need to be thawed at least 1 week before using them for differentiation; therefore, this step must be completed in advance.

5. Coat a dish with 0.1% gelatin for at least 20 min.

***Note:*** Choose an appropriate dish with a surface area that matches the cell number. We freeze 500,000 cells per tube and thaw them onto a 60 mm Cellbind dish.

6. Thaw a frozen stock of ESCs.
  a. Resuspend the ESCs in 10 mL warm N2B27 medium.
  b. Centrifuge at 218 × *g* for 4 min using a benchtop centrifuge.
  c. Resuspend the pellet in N2B27 + 2i + LIF medium and plate cells on the coated culture dish.
7. Incubate at 37°C, 5% CO_2_.

***Note:*** Provide fresh N2B27 + 2i + LIF daily.

8. Split cells once ESCs form numerous compact colonies with a clear boundary (usually after 2–3 days).

***Note:*** Prevent overgrowth. When mESCs begin to touch and merge, they will spontaneously differentiate and not perform well in the differentiation assay.

9. Maintain cells in N2B27 + 2i + LIF medium for at least 1 week before using them for the differentiation assay.

## Key resources table

**Table:** 

REAGENT or RESOURCE	SOURCE	IDENTIFIER
**Antibodies**		

Mouse AP2ALPHA	Santa Cruz	Cat# SC-12726
Rabbit LMX1A	Sigma-Aldrich	Cat# HPA030088
Mouse ASCL1	BD Pharmingen	Cat# 556604
Rabbit ATOH1	Proteintech	Cat# 21215-1-AP
Goat SOX2	R&D	Cat# AF2018

**Chemicals, peptides, and recombinant proteins**		

SU8-GM1075 photoresist	Gersteltec	Cat# GM1075
SU8 developer	Gersteltec	Cat# PGMEA
Isopropanol∗	MicroChemicals GmbH	Cat# MIPU1025
Trichloro(1H,1H,2H,2H-perfluorooctyl-)-silane	Sigma-Aldrich	Cat# 448931
Sylgard 184 Silicone 1 kg Elastomer Kit	Biesterfeld Spezialchemie	Cat# G5498840000
0.1% Gelatin in water	STEMCELL Technologies	Cat# 07903
DMEM/F-12, no glutamine	Gibco	Cat# 21331020
Neurobasal (NB) medium	Gibco	Cat# 21103049
Accutase	Gibco	Cat# A1110501
Bovine serum albumin (BSA)	Sigma-Aldrich	Cat# A3156
N-2 supplement (100X)	Gibco	Cat# 17502001
B-27 supplement (50X)	Gibco	Cat# 17504001
GlutaMAX	Gibco	Cat# 35050061
L-glutamine	Gibco	Cat# 25030024
Penicillin-Streptomycin (P-S)	Gibco	Cat# 15140122
2-Mercaptoethanol	Gibco	Cat# 31350010
bFGF	R&D	Cat# 3139–FB–025
ROCK inhibitor Y-27632	Tocris	Cat# 1254
CHIR99021	Axon	Cat# 1386
PD98059	Cell Signaling Technology	Cat# 9900
LIF	Sigma-Aldrich	Cat# ESG1107
BMP4	R&D	Cat# 5020-BP-010
Retinoic acid (RA)	Sigma-Aldrich	Cat# R2625

**Experimental models: Cell lines**		

HM1 mouse embryonic stem cells	Magin et al.^16^	mouse ESC: HM1

**Software and algorithms**		

CAD software (e.g., Adobe Illustrator∗)	Adobe	https://www.adobe.com

**Other**		

Film 9″ × 12″ Photomask∗	JD Photo Data	Highest resolution (∼10 μm)
Transparent glass plate∗	JD Photo Data	125 mm × 125 mm × 2.35 mm
Silicon wafers 100 mm∗	Siegert Wafer	Cat# BW14001
Mixing cups 185 mL suitable for speed mixer∗	Hauschild	PP150-310 mL Cat# 1000005111
Square plastic Petri dish∗	Greiner	Cat# 688102
Flat tweezers	N/A	N/A
Pointed tweezers	N/A	N/A
μ-Slide 2-well ibiTreat	ibidi	Cat# 80286
CellBIND surface, 60 mm	Corning	Cat# 3295
15 mL Falcon tubes∗	Sarstedt	Cat# 62.554.502
Cell counting slides∗	Bio-Rad	Cat# 1450011
Spin coater∗	Polos	Spin150i
Digital programmable hotplate∗	Harry Gestigkeit	1°C resolution, 290 mm × 210 mm
Mask Aligner∗	EVG, Austria	EVG 610 Mask Aligner with mercury lamp
UV filter∗	Omega Optical	PL-360LP 215 mm × 215 mm
Crystallizing dish∗	Duran	140 mm diameter Cat# 213135409 115 mm diameter Cat# 213134901
Vacuum desiccator∗	Duran	NOVUS DN 200 clear
Nitrogen spray gun∗	ipolymer.com	Nitro-4
Nitrogen spray gun∗	https://cleanroomworld.com/	with filter and Luer lock attachment TA-Nitro-3
Benchtop ultrasonic cleaner∗	Powersonic	P1100 with basket
Inspection microscope∗	Nikon	Nikon Eclipse L200N with 5x/10x objective
Microscope camera∗	Optoteam	G5
SpeedMixer∗	Hauschild	DAC 150
Desiccator∗	Bel-Art	230 mm Plate Size Cat# 999320237
Spin coater∗	Laurell	WS-650-23
Micrometer screw gauge	Mitutoyo	1 μm resolution micrometer
Needle, blunted end∗	McMaster Carr	27G Luer lock
Convection incubator/oven reaching 80°C∗	Binder	ED 56
Inverted microscope∗	Olympus	CKX41
Vacuum pump∗	Vacuubrand	MZ 2C NT
Chemical fume hood∗	Wesemann	N/A
Benchtop centrifuge∗	VWR	Cat# 521-1752
TC20 automated cell counter∗	Bio-Rad	Cat# 1450102
Confocal microscope∗	Nikon	CSU-W1

∗ = or equivalent.

## Materials and equipment

**Table:** Wash medium

Reagent	Final concentration	Amount
DMEM/F12	50%	100 mL
Neurobasal Medium	50%	100 mL
20% BSA	0.08%	0.8 mL

Store at 4°C for up to 1 month.

**Table:** N2B27 medium

Reagent	Final concentration	Amount
DMEM/F12	48%	96 mL
Neurobasal Medium	48%	96 mL
20% BSA	0.08%	0.8 mL
N2 Supplement	1x	1 mL
B27 Supplement	1x	2 mL
P-S	100 U/mL	2 mL
Glutamax	2 mM	2 mL
2-Mercaptoethanol	0.1 mM	0.2 mL

Store at 4°C for up to 10 days.

**Table:** N2B27 + 2i + LIF medium

Reagent	Final concentration	Amount
N2B27	99.87%	50 mL
CHIR99021 10 mM	3 μM	15 μL
PD98059 20 mM	1 μM	2.5 μL
LIF	1,000 U/mL	50 μL

Store at 4°C for up to 3 days.

**Table:** Desiccator medium

Reagent	Final concentration	Amount
DMEM/F12	50%	50 mL
Neurobasal Medium	50%	50 mL

Store at 4°C for up to 1 month.

## Step-by-step method details

### Part 1: Mold preparation

**Timing: 1 day**

For mold fabrication, a silicon wafer coated with photoresist (SU8) is exposed to UV light through a photomask to activate the SU8 in a specific pattern. Subsequent baking solidifies the pattern, and unexposed regions are dissolved in the developer to leave the desired mold pattern. The mold is then coated with a hydrophobic silane layer to prevent unwanted adhesion of PDMS during stencil production.

***Note:*** There are numerous steps. Familiarize yourself with the workflow.

***Note:*** For experienced users, the mold preparation step should be highly reproducible. Difficulties that users may encounter are covered in the troubleshooting section.

#### Prepare the photomask

***Note:*** The photomask is a 0.18 mm thick polyester film with a photographic emulsion on one side. In this step, we cut and tape it to a glass plate.

**Figure 2:**
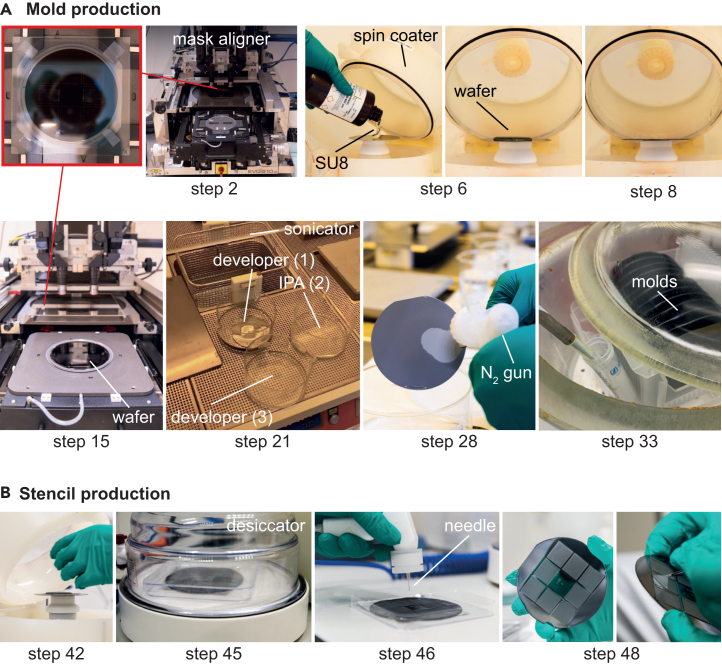
Illustrations of critical steps of the protocol (A) Mold production. The photomask is taped onto the glass plate (outlined with red box) and placed in the mask aligner (step 2). The wafer is spin coated with SU-8 (steps 6–8) and loaded onto the mask aligner (step 15) and exposed after baking (not shown). After that, the pattern is developed (steps 21–27). The mold is silanized (steps 32–34). (B) Stencil production. The mold is spin coated with PDMS (steps 42–43) and degassed (step 45). A needle attached to an air gun is used to expose posts (step 46). After baking (not shown), stencils are peeled off the molds using tweezers (step 48).

***Note:*** The mask aligner chuck and tray can be used as a reference to center the mask relative to the glass plate.

1. Before use, cut out a single 4″ × 4” (101.6 × 101.6 mm^2^) photomask pattern from the film.
2. Tape the selected photomask flat onto the center of the transparent 5″ × 5” (127 × 127 mm^2^) glass plate with the printed surface facing away from the glass and facing the wafer (Figure 2A).

**CRITICAL:** Wear double gloves to avoid exposing the developer or photoresist to your fingers.

#### Dry silicon wafer

3. Set the hot plate to 110°C and bake the wafers for 5 min to remove moisture and improve adhesion.
4. Cool the wafers to 20°C–25°C before use.

#### Spin coat the wafer with SU8 photoresist

5. Center the wafer on the chuck of the spin coater and apply a vacuum to hold it in place.
6. Dispense SU8-GM1075 onto the center of the wafer by pouring from the bottle until approximately 40%–50% of the diameter is covered (Figure 2A).
7. Program the spin coater to spin at 500 rpm with an acceleration of 100 rpm/s for 110 s, followed by 900 rpm for 1 s with an acceleration of 400 rpm/s.
8. When the wafer is stationary, release the vacuum and move the wafer to a level surface at 20°C–25°C for a minimum of 10 min until the photoresist is leveled (Figure 2A).

***Note:*** Air bubbles in the SU8 should be removed using a razor or needle to reduce defects in the final mold.

9. Transfer the wafer to the hot plate.
10. Ramp the temperature to 40°C over 5 min.
11. Bake the wafer at 40°C for 30 min.
12. Ramp the temperature to 120°C over 20 min.
13. Bake the wafer at 120°C for 50 min and then cool to 20°C–25°C.

***Note:*** At this step, the mask aligner should be turned on to warm up the mercury lamp for at least 30 min before use.

#### Photolithography of pattern

In this step, a mask aligner is used to position the photomask over the SU8-coated wafer. UV light through the photomask patterns the SU8.

14. Use the settings outlined in Table 1 for the mask aligner (EVG 610).

**Table 1:** Mask aligner settings

Mask aligner setting	Value
Process	Man. Top Side
Process Mode	Transparent
Exposure Mode	Constant Dose
Contact Mode	Soft Contact
Mask holder	Size 5 inch
Chuck	Size 4 inch
Separation	10 μm
Thickness Mask	2.3 mm
Thickness Substrate	1 mm
Thickness Resist	300 μm
Exposure, Dose	1500 mJ/cm^2^
Process	600 mbar
WEC	300 mbar
Exposure	600 mbar
15. Follow the instructions on the mask aligner to load the glass plate with the photomask and SU8-coated wafer (Figure 2A).
16. Add the UV filter (PL-360LP) to the light path.

***Note:*** The UV filter gives the structures more vertical side walls and stops defects on the top edges of the SU8 (T-topping).

***Note:*** The soft contact mode on the mask aligner must be used to avoid distortion of the SU8.

17. Expose SU8 as defined in the mask aligner settings above. Troubleshooting problem 3.
18. After exposure, place the wafer on a hot plate at 20°C–25°C and set the temperature to 95°C. After the hot plate has warmed to 95°C, bake wafers for 2.5 h.
19. Allow wafers to cool to 20°C–25°C.

#### Develop the photoresist to reveal the pattern

Following UV exposure and baking, the SU8 is ready for development. In combination with sonication, the developer dissolves SU8 that was not exposed to UV light.

**Figure 3:**
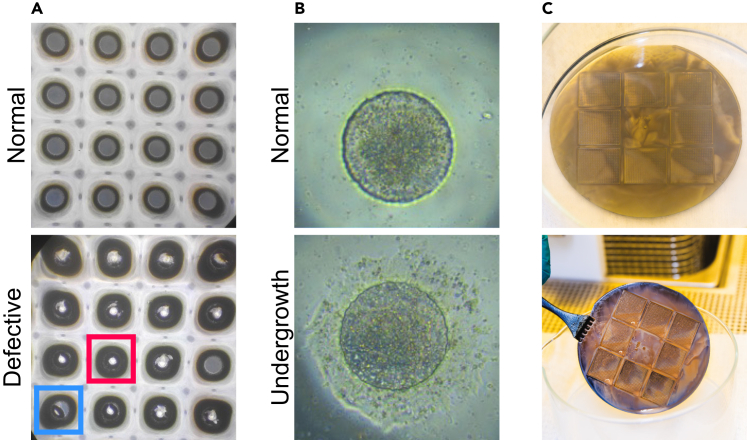
Troubleshooting (A) Potential stencil defects. *Normal:* Bright-field image of a good quality stencil with clearly defined holes. *Defective:* A bad quality stencil with multiple defects. The blue square shows a loose cap, and the red square shows a non-through hole; these present as a darker-rimmed hole with a light center as they reflect the condenser of the microscope. (B) Stencil attachment problems. *Normal:* Bright-field image of a colony before stencil removal on Day 2 (step 72). The colony is well contained within the well boundaries. *Undergrowth:* Bright-field image of a colony that has grown under the stencil, resulting in a poorly defined border. This type of defect can occur in up to 30% of the wells of a stencil. See Troubleshooting problem 5 for details. (C) Residues of SU-8 after mold development (step 24). See Troubleshooting problem 4 for details.
25. Transfer the mold to dish 3 (with clean SU8 developer) to clean the residues by gently swirling the liquid around the wafer. Troubleshooting problem 4.
26. After all residues are dissolved, transfer the mold back to the isopropanol dish (dish 2) to check again.
27. If no white precipitate forms, wash once more in SU8 developer (dish 3). Otherwise, repeat steps 25 and 26.

***Note:*** Usually, it is not necessary to repeat the wash step.

20. Prepare 3 crystallizing dishes of 140 mm diameter. Pour SU8 developer into dishes 1 and 3. Fill dish 2 with isopropanol (approximately 1 cm high).
21. Transfer the mold into dish 1 (with SU8 developer) (Figure 2A).
22. Place the dish with the mold in the ultrasonic cleaner.
23. Sonicate for 6 min at power 9 at 20°C–25°C.
24. Remove the mold from the ultrasonic cleaner and place it into the isopropanol dish (dish 2) to check for residues. If there are residues, they will be visible as dark waves (Figure 3C).

28. Remove the mold from the developer. Hold the wafer with tweezers and blow the residual developer off the back of the wafer using the N_2_ gun (Figure 2A).

***Note:*** The back of the wafer must be dry to ensure proper vacuum seal on the chuck of the spin coater.

29. Mount the mold on the spin coater and spin at 3000 rpm for 30 s to dry the top.
30. Bake for 5 min at 135°C on a hot plate to make SU8 permanent and avoid cracks in the material.
31. Check the mold under an inspection microscope for any irregularities in post diameters and inspect for damage.

#### Silanization of the mold

Silanization of the mold adds a hydrophobic coating, which prevents the PDMS from sticking to the mold during stencil production.

***Note:*** Trichloro-(1H,1H,2H,2H-perfluorooctyl)-silane reacts violently with water and is highly corrosive. Use robust gloves and a fume hood.

32. In a fume hood, place the molds in a desiccator connected to a vacuum pump.
33. Pipette 20 μL of Trichloro-(1H,1H,2H,2H-perfluorooctyl)-silane into a small tube and place it inside the desiccator without the lid (Figure 2A). Eject the tip into the tube.
34. Close the desiccator and pull a vacuum of at least 100 mbar to vaporize the Trichloro-(1H,1H,2H,2H-perfluorooctyl)-silane. Troubleshooting problem 2.

***Note:*** Reaching 100 mbar may take several minutes, depending on the specific vacuum pump used.

35. Completely seal the vacuum chamber of the desiccator.
36. After 1 h, let the air back in slowly.
37. Cap the tube and dispose of it carefully.
38. To store the wafers, put them in separate square dishes lined with aluminum foil sheets at the bottom.

### Part 2: PDMS stencil production

**Timing: minimum 3 h**

In this part, we use the molds manufactured in the previous section to cast the PDMS stencils (Figure 2B). We spin-coat the mold with a layer of PDMS, gently blow with N_2_ to expose posts and ensure through holes in PDMS layer, then bake the PDMS to solidify it.

***Note:*** The quality of stencils is mostly determined by the quality of the mold. Fresh molds yield high-quality stencils that contain > 80% through holes with smooth edges. Repeated use of molds decreases the yield and quality of stencils. Therefore, molds should be discarded after 5 cycles of PDMS stencil production. To reuse molds, the residual PDMS needs to be carefully removed using tweezers.

39. Use the Sylgard Elastomer 184 kit to prepare a PDMS mixture as directed. For this, mix the provided elastomer and curing agent in a 10:1 w/v ratio in a SpeedMixer mixing cup. Prepare approximately 10 g of PDMS mixture per wafer.

***Note:*** Weigh the elastomer component first and use a plastic Pasteur pipette to measure the volume of the curing agent accurately.

***Note:*** It is practical to process multiple wafers at the same time. We usually make 12 in parallel. Scale up the mixture as necessary.

40. Seal the lid and mix at 2000 rpm for 2 min in a SpeedMixer.
41. Center the mold on the chuck of the spin coater and apply a vacuum.
42. Dispense approximately 10 mL of PDMS mixture onto the center of the mold until approximately 50%–70% of the diameter is covered (Figure 2B).
43. Spin at 600 rpm for 30 s in a spin coater (for this, program the spin coater to 600 rpm for 40 s with an acceleration of 60 rpm/s).
44. Release the vacuum and remove the mold. Take care to avoid PDMS drops from the lid of the spin coater entering into the vacuum chuck. Place the mold in a square petri dish on an aluminum foil sheet.
45. Using a desiccator, apply a vacuum to the coated mold for 1 min to degas the PDMS (Figure 2B). Troubleshooting problem 3.
46. Connect a blunted 27G needle to an air gun and blow at 10 psi to expose posts that PDMS covers (Figure 2B). Hold the needle approximately 1–1.5 cm away from the mold. Blow over the mold in a regular grid pattern (up-down, left-right). Do two rounds on all molds. Troubleshooting problem 1.

***Note:*** The flow rate needs to be adjusted carefully to prevent the scattering of too much PDMS while ensuring exposure of the top of the pillars at the same time. Check whether all posts are exposed under the microscope and repeat step 46 if necessary.

47. Bake the PDMS-covered molds in square dishes at 80°C for 2 h. Troubleshooting problem 5.
48. After cooling to 20°C–25°C, gently peel the stencils from a corner and place them onto a clean surface (e.g., a square petri dish).
49. Inspect the stencils under the microscope. Stencils with less than 75% through holes or jagged holes should be discarded (Figure 3A).

***Note:*** The final PDMS stencils should be approximately 210 μm thick when measured with a micrometer screw gauge.

50. Place the stencils flat into a square petri dish or similar closed clean container for storage.

### Part 3: Differentiation of spinal cord progenitors from mouse ES cells on stencils

**Timing: minimum 3 days**

In the following steps, stencils are placed on coated ibiTreat dishes. Cells that are pre-differentiated for 24 h in bFGF^1^ are plated on the stencils and attached to the dish within the stencil holes. Stencils are then removed and cells are exposed to further differentiation cues.

***Note:*** Here, we use stencils in conjunction with a protocol for differentiation of mouse dorsal posterior neural tube cells, which is described in detail in Lehr et al.^1^

***Note:*** Successful completion of this step depends on cell culture experience, well-maintained cells, and good sterile technique.

#### Day 0: Coat dishes and sterilize stencils

**Timing: 10 min to 1 h**

51. Coat the required number of 2-well μ-slide ibiTreat dishes with 0.1% gelatin in H_2_O.

***Note:*** Do not use gelatin in PBS, as this will form crystals and interfere with proper stencil attachment.

52. Leave the coated dishes in the incubator at 37°C for a minimum of 3 h.
53. As a precaution, sterilize stencils using UV light in a laminar flow hood for 30 min.

***Note:*** To reduce contamination risk in later steps, clean and sterilize tweezers and square petri dishes (with lids open) by exposure to UV light in the laminar flow hood for 30 min. Ideally, use a desiccator dedicated to this step that does not come in contact with chemicals and is maintained in a sterile condition.

#### Day 1: Plate cells onto stencils

**Timing: 2–3 h**

54. Aspirate the gelatin and leave dishes to dry at an angle for 30–40 min before placing stencils. Troubleshooting problem 5, problem 6.
55. Carefully place the stencils into the dishes using sterile forceps/tweezers. Troubleshooting problem 5.

***Note:*** Ensure that the stencils are placed straight and without bulges and are not touching the edges of the dish, as this will lead to detachment.

***Note:*** Due to surface tension, air will be trapped in the holes of the stencil.

56. To remove the air bubbles in the holes of the stencil, add 2 mL of Desiccator medium to the wells.

**CRITICAL:** Media at this step should not contain BSA as this will act as a foaming agent.

57. Place ibidi dishes (with lids off) inside sterile square plastic petri dishes. Five ibidi dishes can be placed inside one square petri dish. Close the lids of the square petri dishes and stack them.
58. Wipe the desiccator with ethanol and place the stack of petri dishes into the desiccator.
59. Apply a vacuum for approximately 1 min.
60. Release vacuum. Most bubbles should be removed. The dishes are now ready to use.
61. To prepare the cells, start by rinsing them with PBS.
62. Dissociate cells to a single-cell suspension using Accutase (1 mL per 10 cm dish).
63. Collect the cells in a 15 mL falcon tube in a total of 10 mL Wash medium.
64. Centrifuge cells at 218 × g for 4 min using a dedicated cell culture centrifuge.
65. Aspirate supernatant and resuspend the cell pellet in 10 mL Wash medium.
66. Gently mix and load 10 μL of the cell suspension into the counting slide chamber for automated counting. Count cells in duplicates.
67. Centrifuge cells again at 218 × g for 4 min.
68. Resuspend cells at 1.25–1.5 million cells per mL in N2B27 medium supplemented with 10 ng/mL bFGF + 10 μM Y-27632 ROCK inhibitor.
69. Aspirate the 1:1 medium from the ibidi dishes and add 2 mL of cell suspension per well. Let the cells settle for approximately 3 h in the incubator.

***Note:*** Be careful not to touch the stencil with the aspirator tip when removing the media as this can lead to detachment.

70. Wash cells twice with wash buffer to remove non-attached cells. Troubleshooting problem 6.

***Note:*** Ensure that all floating cell clumps are removed, as those would reattach to your 2D colonies.

71. Add 2 mL N2B27 medium supplemented with 10 ng/mL bFGF and return the cells to the incubator.

#### Day 2: Remove stencils

**Timing: less than 30 min**

72. Using dry sterile forceps, pinch one corner of the stencil and peel it off carefully. Troubleshooting problem 6.

***Note:*** Stencils are removed while the cells are still in media. Be careful not to disturb the attached colonies when peeling the stencil. Forceps with slightly bent tips work better for this as they naturally hook under the stencil.

73. Wash the cells with Wash medium.
74. Add N2B27 medium supplemented with 10 ng/mL bFGF and 5 μM CHIR and culture for 24 h to obtain neuromesodermal progenitors.
75. After this point, continue with specific neural differentiation protocol. For full dorsal neural differentiation protocol, see Methods in Lehr et al.^1^

## Expected outcomes

Mold production should yield molds with uniformly shaped and spaced pegs on top of the silicon wafer. Any defects in the mold will be transferred onto the stencils and subsequently affect the uniformity of the colonies.

Stencil production should result in PDMS stencils with uniform holes with clean, well-defined edges (Figure 3A). When using newly made molds, stencil production is highly efficient. Good quality stencils will result in sharp-edged circular colonies that are restricted to the well boundaries and do not grow or spread under the stencil (Figure 3B). Holes or clumps inside the patterned colonies result from suboptimal seeding densities.

**Figure 4:**
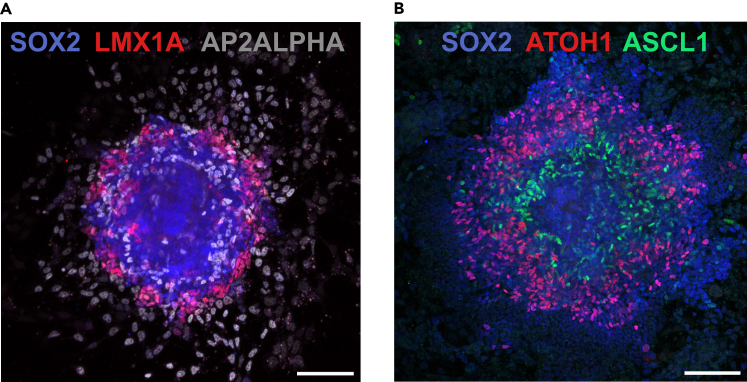
Self-organized patterns of neural crest and dorsal neural progenitor differentiation Successful differentiation protocol results in self-organized radial patterns of dorsal neural tube cell types, such as the ones shown here. Immunofluorescence staining of dorsal neural tube progenitor domains. (A) Colony at t = 48 h after the addition of 100 nM RA and 0.5 ng/mL BMP4. Immunostaining against pan-neural progenitor marker SOX2, roof plate marker LMX1A, and neural crest marker AP2ALPHA. (B) Colony at t = 96 h after the addition of 100 nM RA and 0.5 ng/mL BMP4. Immunostaining against pan-neural progenitor marker SOX2, dp1 marker ATOH1, and dp3-5 marker ASCL1. For the specific details of neural differentiation, see Lehr et al.^1^ Scale bars, 100 μm.

Successful completion of the differentiation protocol should result in a high proportion (50%–80%) of colonies with a radial pattern of expression of dorsal neural tube cell types. After stencil removal and subsequent 24 h treatment with RA and BMP4, as described in Lehr et al.,^1^ neural crest cells are observed migrating outside of the colony, while roof plate cells are localized at the colony periphery. Within the colony, neural progenitor domains dp1 to dp6 form in their characteristic order^17^ from most dorsal at the periphery towards ventral in the colony center (Figure 4).

## Limitations

Using this method, it is challenging to achieve wells smaller than 300 μm. This is because a reduction of the post diameter produces a high height-to-width aspect ratio of the posts, which makes the mold more fragile.

The highest quality stencils are derived from the middle positions along the edges of the mold (Figure 1: A2, B1, B3, and C2), while corner stencils (Figure 1: A1, A3 and C1, C3) degrade in quality faster with repeated use. The center position cannot be used for stencils due to limitations in spin coating efficacy in the center of the wafer.

Stencils will not remain attached to the dish surface through successive cycles of media changes, therefore, for long-term confinement of cells, other methods might be preferable.

## Troubleshooting

### Problem 1

Too few through holes in stencils.

Common defects are non-through holes (Figure 3A, red square). A related issue is caps (small circles of partly attached PDMS from the tops of pegs, Figure 3A, blue square); these can remain attached to the holes or become loose. Loose caps can stick to the bottom of stencils and prevent attachment of the stencil to the coated cell culture dish.

### Potential solution 1

• Stencil defects usually arise at the blowing step when the pillars remain covered by PDMS (step 46, Figure 2B). Inspecting the PDMS-covered molds under the microscope and repeating the blowing step if necessary is important. Too much pressure can result in the spreading of PDMS to neighboring stencils.
• Caps are likely to result from poor clearing of the PDMS from the mold when the mold is reused. Carefully clean the residual PDMS off the molds using sharp tweezers for the larger pieces and the air gun.

### Problem 2

Holes in stencil have rough edges, resulting in irregular colony shapes.

Rough stencil edges can result from the bonding of PDMS to the SU8 due to poor silanization (steps 32–37). Similar to caps (see problem 1), rough edges can also result from poor clearing of the PDMS from the mold when the mold is reused.

### Potential solution 2

• After successful silanization, the surface of the mold should be highly hydrophobic. Complete coating can be assessed by pipetting a 5 μL drop of water on the mold surface and checking the contact angle of the drops is greater than 90 degrees.

### Problem 3

Distortions in stencil pattern.

Distortions in the stencil pattern can have multiple causes. Air bubbles trapped in the PDMS can lead to deformations and size variations of the holes. Another potential source is misalignment of the sandwich for the mask aligner, depending on your equipment.

### Potential solution 3

• Air bubbles in the PDMS can be removed by repeated vacuum treatment (step 45).
• For the sandwich (steps 15–17), ensure correct order (glass plate, photomask with printed side away from glass and towards wafer, wafer) and alignment of all components. Incorrect order could lead to distortions.
• Make sure not to fold or scratch the photomask.

### Problem 4

Rough mold surface.

The surface of the molds needs to be clean and smooth to ensure flat stencils that stick well. Uneven mold surface can result from insufficient development of the SU8 photoresist (steps 20–27).

### Potential solution 4

• Inspect the molds after washing in isopropanol (step 24) under the microscope. If you detect uneven surfaces or residues, repeat the sonication step (step 23) or increase the sonicator power or duration for subsequent wafers.

### Problem 5

Poor stencil attachment.

Poor stencil attachment to the dish causes cells to grow under the stencil and distorted colony borders, or even detachment and subsequent floating of the stencil.

### Potential solution 5

• Dishes must be dry and free of crystals (e.g., caused by salts in the coating solution) and other residues for the stencils to stick properly (step 54). Use gelatin in water, and carefully inspect the wells for gelatin drops before you place the stencil. Dry the dishes at an angle to ensure there are no residues from the dried liquid droplets in the center of the wells.
• Make sure not to touch the bottom of the stencil with gloves during production or handling in cell culture, and store the stencils on a clean surface (e.g., a petri dish).
• Place the stencil flat into the center of the well and make sure the edges are sticking down and not touching the well walls (step 55). When aspirating media from the wells, be careful not to touch the stencil with the aspirator tip.
• The baking time of the stencil (step 47) needs to be carefully adjusted to ensure successful polymerization while retaining adhesiveness.

### Problem 6

Poor cell attachment or colony pattern.

Plating, attachment, and too few or too many cells can result in irregular colony shapes and colonies that grow or pattern poorly.

### Potential solution 6

• Make sure to properly resuspend the cells to have reliable counts and sufficiently high cell numbers.
• Overdrying of dishes after the coating (step 54) can cause poor cell attachment.
• Too short of a vacuum treatment can result in blocked holes, preventing cells from attaching to the dish surface.
• Inefficient washing (step 70) can cause excess cells in the media to clump and reattach to the dish surface after stencil removal.
• Avoid applying shear forces when removing the stencil (step 72), as this can result in colony detachment or distortions.

## Resource availability

### Lead contact

Further information and requests for resources and reagents should be directed to the lead contact, Anna Kicheva (anna.kicheva@ist.ac.at).

### Technical contact

Questions about the technical specifics of performing the protocol should be directed to the technical contact, Jack Merrin (jack.merrin@ist.ac.at).

### Materials availability

This study did not generate new unique reagents.

### Data and code availability

This study did not generate datasets or code.

## References

[1] Lehr S., Brückner D.B., Minchington T.G., Greunz-Schindler M., Merrin J., Hannezo E., Kicheva A. (2024). Self-organized pattern formation in the developing mouse neural tube by a temporal relay of BMP signaling. Dev. Cell.

[2] Minchington T., Lehr S., Kicheva A. (2023). Control of tissue dimensions in the developing neural tube and somites. Curr. Opin. Syst. Biol..

[3] Blin G. (2021). Quantitative developmental biology in vitro using micropatterning. Dev.

[4] Gjorevski N., Nikolaev M., Brown T.E., Mitrofanova O., Brandenberg N., DelRio F.W., Yavitt F.M., Liberali P., Anseth K.S., Lutolf M.P. (2022). Tissue geometry drives deterministic organoid patterning. Science.

[5] Warmflash A., Sorre B., Etoc F., Siggia E.D., Brivanlou A.H. (2014). A method to recapitulate early embryonic spatial patterning in human embryonic stem cells. Nat. Methods.

[6] Chhabra S., Liu L., Goh R., Kong X., Warmflash A. (2019). Dissecting the dynamics of signaling events in the BMP, WNT, and NODAL cascade during self-organized fate patterning in human gastruloids. PLoS Biol..

[7] Morgani S.M., Metzger J.J., Nichols J., Siggia E.D., Hadjantonakis A.K. (2018). Micropattern differentiation of mouse pluripotent stem cells recapitulates embryo regionalized cell fate patterning. eLife.

[8] Sahni G., Yuan J., Toh Y.C. (2016). Stencil micropatterning of human pluripotent stem cells for probing spatial organization of differentiation fates. J. Vis. Exp..

[9] Falconnet D., Csucs G., Grandin H.M., Textor M. (2006). Surface engineering approaches to micropattern surfaces for cell-based assays. Biomaterials.

[10] Théry M. (2010). Micropatterning as a tool to decipher cell morphogenesis and functions. J. Cell Sci..

[11] Duval N., Vaslin C., Barata T.C., Frarma Y., Contremoulins V., Baudin X., Nedelec S., Ribes V.C. (2019). BMP4 patterns Smad activity and generates stereotyped cell fate organization in spinal organoids. Development.

[12] Andrews M.G., del Castillo L.M., Ochoa-Bolton E., Yamauchi K., Smogorzewski J., Butler S.J. (2017). BMPs direct sensory interneuron identity in the developing spinal cord using signal-specific not morphogenic activities. eLife.

[13] Gouti M., Tsakiridis A., Wymeersch F.J., Huang Y., Kleinjung J., Wilson V., Briscoe J. (2014). In Vitro Generation of Neuromesodermal Progenitors Reveals Distinct Roles for Wnt Signalling in the Specification of Spinal Cord and Paraxial Mesoderm Identity. PLoS Biol..

[14] Choi J.H., Lee H., Jin H.K., Bae J.S., Kim G.M. (2012). Micropatterning of neural stem cells and Purkinje neurons using a polydimethylsiloxane (PDMS) stencil. Lab Chip.

[15] Folch A., Jo B.-H., Hurtado O., Beebe D.J., Toner M. (2000). Microfabricated elastomeric stencils for micropatterning cell cultures. J. Biomed. Mater. Res..

[16] Magin T.M., McWhir J., Melton D.W. (1992). A new mouse embryonic stem cell line with good germ line contribution and gene targeting frequency. Nucleic Acids Res..

[17] Alaynick W. a, Jessell T.M., Pfaff S.L. (2011). SnapShot: spinal cord development. Cell.

